# Diplopia due to Dacryops

**DOI:** 10.1155/2013/549487

**Published:** 2013-12-04

**Authors:** Rahmi Duman, Reşat Duman, Mehmet Balcı

**Affiliations:** ^1^Department of Ophthalmology, Dr. Abdurrahman Yurtaslan Oncology Training and Research Hospital, Yenimahalle, 06105 Ankara, Turkey; ^2^Department of Ophthalmology, Emirdağ Hospital, Emirdağ, 03600 Afyon, Turkey

## Abstract

Dacryops is a lacrimal ductal cyst. It is known that it can cause globe displacement, motility restriction, and proptosis because of the mass effect. Diplopia due to dacryops has not been reported previously. Here, we present a 57-year-old man with binocular horizontal diplopia that occurred during left direction gaze due to dacryops.

## 1. Introduction

Dacryops is a rare clinical entity characterized by cyst formation in the lacrimal gland. Although it can arise in any location where lacrimal gland tissue is present, it usually presents as painless swelling under the lateral aspect of the upper eyelid. Although, in many cases, there is no a predisposing factor, in some cases, it may be preceded by local trauma or inflammation and also there is a reported case that occurred after chemical injury [1]. It is thought that trauma or inflammation causes ductal wall damage and weakened lacrimal ductal contractility, resulting in distention of the duct wall and consequent cyst formation [1–3].

To best of our knowledge, diplopia due to dacryops has not been reported previously.

## 2. Case Report

A 57-year-old man was referred to the ophthalmology department with binocular horizontal diplopia that was present for 2 weeks. The diplopia occurred in only left direction of gaze and had progressed over the past days. There was no associated pain, erythema, or discharge. And past medical history of the patient was negative for any orbital trauma or infection.

During ophthalmological examination, corrected visual acuity was 20/20 OD and 20/20 OS. The external examination revealed no inflammation, mass, or proptosis. There was no difference between the right and left eye in the exophthalmometry evaluation and extraocular movements were full in both eyes. At eyelid eversion examination of the right eye, small cystic mass was identified on the anterior aspect of the palpebral lobe of the lacrimal gland. The cyst appeared smooth and translucent and was attached to the underlying conjunctiva.

**Figure 1:**
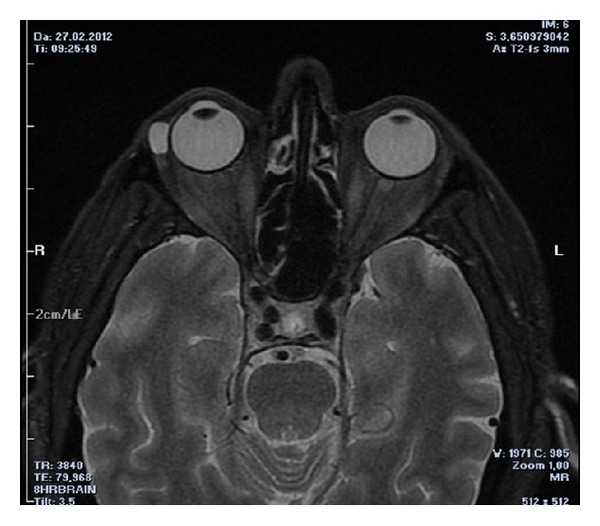
Magnetic resonance image of dacryops.

Magnetic resonance (MR) imaging revealed a 11 × 6.5 mm well-circumscribed cystic lesion adjacent to the lateral rectus and contiguous with the inferior palpebral lobe of the lacrimal gland (Figure 1). The globe and the remaining lacrimal gland tissue were normal and there was no associated periorbital or soft-tissue inflammation.

A lateral orbitotomy was performed. During excision, the cyst was ruptured and thin, watery, clear fluid gushed out. Postoperatively, the patient's diplopia was resolved.

Histopathological examination of the surgical specimen revealed a cystic space lined by columnar epithelial cells consistent with ductal origin and this finding was compatible with the diagnosis of dacryops.

## 3. Discussion

Dacryops, lacrimal ductal cyst, usually appears as a a slowly growing well-circumscribed translucent cyst in the palpebral lobe of the lacrimal gland and presents as painless swelling in the lateral portion of the upper eyelid. In the setting of superimposed infection, rapid growth can be observed. Mass effect of enlarging cyst can displace the globe, restrict motility, and cause proptosis [3, 4].

Etiopathogenesis of dacryops is not clearly known, but it is speculated that preceding trauma or inflammation results in ductal wall damage. Decreased contractility and lacrimal gland hypersecretion both cause dilatation of the ductal wall and subsequent cyst formation [1–3].

In differential diagnosis of dacryops, other benign and malign orbital tumours such as pleomorphic adenoma, adenoid cystic carcinoma, mucoepidermoid carcinoma, pleomorphic adenocarcinoma, lymphoma, pseudolymphoma, and metastasis and developmental cysts such as dermoid and epidermoid cysts must be considered. MR imaging is a preferably used imaging modality to assess orbital tumours because of its advantage of better soft tissue evaluation than that of computerized tomography imaging. Dacryops can be easily diagnosed by MR imaging as a well-defined, small cystic lesion and appears to be hypointense on T1-weighted and hyperintense on T2-weighted imaging [5], but the definitive diagnosis is made by histopathological examination. Dacryops is histopathologically characterized by cystic spaces lined by ductal epithelium and surrounded by glandular tissue and dilated ducts [1, 2]. In our patient, both MR imaging findings and histopathological examination were compatible with dacryops.

To the best of our knowledge, diplopia case due to dacryops has not been reported previously. In our patient, we thought that diplopia during left lateral gaze occurred due to dacryops because after detailed ophthalmological examination and MR imaging no other cause of diplopia was found and also diplopia was resolved after the operation. We suggested that diplopia in this case may be developed because of the mass effect of dacryops on lateral rectus muscle.

In conclusion, although dacryops is a rare ophthalmological entity, it must be included in the differential diagnosis of orbital masses. And clinicians and radiologists should be aware of its variable presentations such as diplopia in our case.
